# Morphological and molecular datasets for *Kaempferia* species

**DOI:** 10.1016/j.dib.2018.10.097

**Published:** 2018-10-27

**Authors:** Catherine Labrooy, Thohirah Lee Abdullah, Johnson Stanslas

**Affiliations:** Universiti Putra Malaysia, Malaysia

## Abstract

This study compared morphological and molecular data for identification of *Kaempferia* species. Each species was deposited in Institute of Bioscience (IBS), Universiti Putra Malaysia (UPM) as voucher specimens and ITS sequences of each species deposited in NCBI (https://www.ncbi.nlm.nih.gov/) as GenBank accessions. DNA was extracted using a modified CTAB method and PCR amplification was completed using Internal Transcribed Spacer (ITS4 and ITS5) markers. PCR amplification of products were viewed under gel electrophoresis. Sequencing was performed and sequence characteristics of ITS rDNA in *Kaempferia* is shown. Qualitative and qualitative scoring of morphological characters and measuring techniques for *Kaempferia* species are included. In addition, a brief review of molecular markers used in phylogenetic studies of Zingiberaceae is included in this dataset.

**Specifications table**

**Table:** 

Subject area	*Biology*
More specific subject area	*Plant taxonomy*
Type of data	*Table, image, figure*
How data was acquired	*PCR amplification using Thermocycler (Biometra), Gel imaging on Alpha Innotech imaging system (Alpha Innotech, San Leandro, CA, USA), Sequence analysis using Clustal X, MEGA 6 and Phylogenetic Analysis Using Parsimony (PAUP) ver. 4.0 Beta 10*
Data format	*Raw and analyzed*
Experimental factors	*DNA was extracted using modified CTAB method*
Experimental features	*DNA barcoding using ITS markers*
Data source location	*University Putra Malaysia, Serdang, Selangor, Malaysia (GPS coordinates 3°0׳26"N 101°42׳16"E)*
Data accessibility	*Data available within article*[1]

**Value of the data**

• ITS4 and ITS5 DNA barcode regions of selected *Kaempferia* species.
• Key morphological characters include petiole length, leaf parameters, rhizome colouring, and plant habit.
• Qualitative and qualitative scoring of morphological characters and measuring techniques for *Kaempferia* species.

## 1 Data

Two datasets are described and compared. Morphology data and molecular data from ITS markers show taxonomic congruence and both can be used to discriminate among closely related *Kaempferia* species.

## 2 Experimental design, materials, and methods

### 2.1 Taxon sampling

Twenty-one accessions of *Kaempferia*, representing six species and one cultivar were included in this study. Three samples were used for each plant. The samples were authenticated by Dr. Shamsul Khamis and voucher specimens of these plants have been deposited at Institute of Bioscience (IBS), Universiti Putra Malaysia (UPM) (Table 1). The rhizomes of all plant samples were cultivated for at Field 2, UPM (GPS coordinates 3°0׳26"N 101°42׳16"E). Cultivated plants were accessed for morphological characters and used for genomic DNA extraction.

**Table 1:** Voucher specimen numbers for *Kaempferia* species deposited in Institute of Bioscience (IBS), Universiti Putra Malaysia (UPM) and GenBank accession number for ITS sequences of each species deposited in NCBI (https://www.ncbi.nlm.nih.gov/).

**Num.**	**Plant species**	**Voucher number**	**GenBank accession number**
1.	*Kaempferia angustifolia* Roscoe.	SK2542/14	KY454694
2.	*Kaempferia galangal* Linn.	SK2539/14	KY454695
3.	*Kaempferia elegans* Wall. ex Baker	SK2540/14	KY454696
4.	*Kaempferia pulchra* Ridl.	SK2541/41	KY454697
5.	*Kaempferia parviflora* Wall. ex Baker (Msia)	SK2537/14	KY454698
6.	*Kaempferia parviflora* Wall. ex Baker (Thai)	SK2538/14	KY454699
7.	*Kaempferia marginata* Carey ex Roscoe.	SK2543/14	KY454700

### 2.2 Molecular methods

Total genomic DNA was extracted from 0.1 g of fresh leaf sample using a modified cetyltrimethyl ammonium bromide (CTAB) extraction protocol [2]. Extracted DNA were quantified using Nanodrop 200c spectrophotometer (Thermo Scientific). Extracted DNA was stored at −20°C until further use. Standard polymerase chain reaction (PCR) were used to amplify the target gene region. The ITS region was amplified (35 cycles of 94 °C for 3 min, 55 °C for 20 sec, 72 °C for 2 min) using primers ITS4 (5׳-TCCTCCGCTTATTGATATGC-3׳) and ITS5 (5’-GGAAGTAAAAGTCGTAACAAGG-3’) [3]. A Thermocycler (Biometra) was used for PCR amplification. Each PCR reaction was in a total volume of 25 µL, containing 15 ng of template DNA, 12.5 µL of 2x Type-it PCR Master Mix (Red-Taq), 1 µL each of 10 µM ITS4 and ITS5, and 8.5 µL of RNase-free water (Qiagen®). Amplification products were separated via electrophoresis on 1.5% (w/v) agarose gels with 1x TBE buffer at 70 V for 75 min, stained with GelRedTM Nucleic Acid Stain and visualized under UV light using Bio-Rad Molecular Imager GelDoc^TM^ XR+ with Image Lab^TM^ Software (Bio-Rad Laboratories, Inc.,USA) (Appendix 1). GeneRuler 100 bp ladder (Fermentas) was used as DNA molecular weight markers. Amplified products were sent for purification and sequencing (First BASE Laboratories Sdn. Bhd.).

### 2.3 Sequence alignment and phylogenetic analysis

ITS sequenced regions were trimmed and aligned using Clustal X with default values (e.g., gap-opening cost = 15) [4], [5] and the resulting alignments were manually checked. Gaps were retained for further analysis. Alignments are available (Appendix 2). Nucleotide diversity (π), estimated values of transition/transversion bias (R), nucleotide substitutions (r) for each nucleotide pair, evolutionary divergence between sequences, and cluster analysis among the *Kaempferia* sequences were estimated using MEGA 6 [6]. For phylogenetic inference, maximum parsimony analysis was performed using Phylogenetic Analysis Using Parsimony (PAUP) ver. 4.0 Beta 10 [7]. Gaps were treated as missing data. The most parsimonious trees were obtained through a heuristic search. A Bootstrap analysis (1000 replicates) was also performed. The phylogenetic tree was re-rooted using the ITS sequence of *Tamijia flagellaris* (K. Schum.) Ridl. (NCBI accession number, AF478797.1) as reported in a previous study [8]. Disregarding indels, nucleotide sequence divergence between pairs of taxa was calculated using a Kimura [9] 2-parameter model. Maximum parsimony analysis was performed using phylogenetic analysis using parsimony (PAUP*) ver. 4.0 Beta 10 (Swofford 2004). Gaps were treated as missing data. The most parsimonious trees were obtained through a heuristic search with tree bisection reconnection (TBR) branch swapping and 10,000 random sequence additions. A Bootstrap analysis (1,000 replicates) with heuristic search was also performed.

### 2.4 Morphological methods

Morphological traits of 21 accessions representing six species were studied at mature plant stage. Twenty-four qualitative and quantitative characteristics were categorized and transformed into scores to represent each category available (Appendix 3). Leaf characters were classified according to Hickey [10] and colour was identified using the Royal Horticultural Society (RHS) colour chart [11], [12] (Appendix 4). The accessions were clustered by the unweighted pair group method using arithmetic average (UPGMA) method which it was indicated as a good cophenetic correlation of the original distance matrices. Multivariate analysis was done by transforming the scores for the presence and absence from each species and analysed using Gower׳s Similarity Index. A dendrogram and coefficient of similarities were produced using MVSP (Multivariate Statistical Programme) to estimate the variability among the *Kaempferia* species.

**Data**

## Figures and Tables

**Fig. A1:**
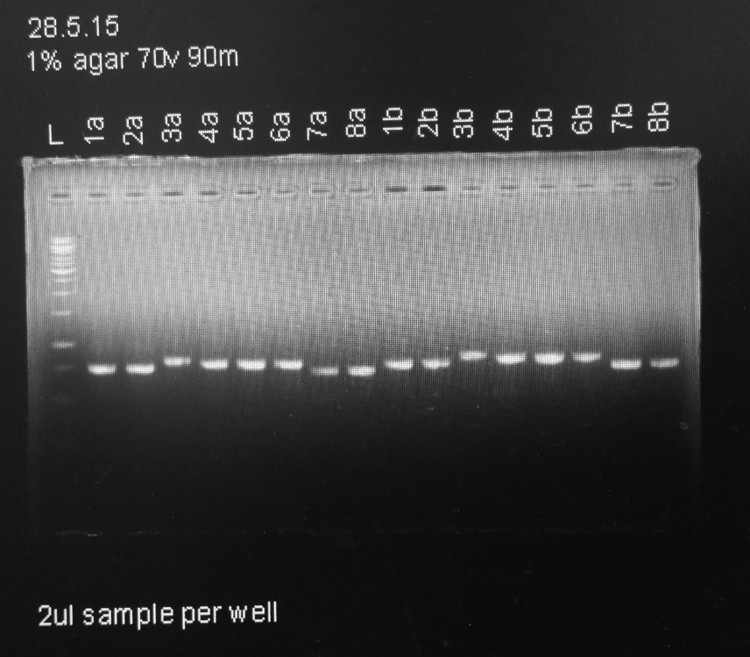
Amplification products separated via gel electrophoresis on 1.5% (w/v) agarose gel with 1x TBE buffer at 70 V for 75 min, stained with GelRed^TM^ Nucleic Acid Stain and visualized under UV light using the Alpha Innotech imaging system. L, ladder; 1a and 1b, *K. parviflora* M; 2a and 2b, *K. parviflora* T; 3a and 3b; *K. galangal*; 4a and 4b; *K. elegans*; 5a and 5b, *K. pulchra*; 6a and 6b, *K. marginata*; 7a and 7b, *K. angustifolia*; 8a and 8b, outgroup *Stahlianthus* (not sequenced).

**Table B1:** Sequence characteristics of ITS rDNA in *Kaempferia*.

**Plant species (Taxon)**	**Sequence length (bp)**	**Sequence**
***K. angustifolia***	707	GATATGCTAAACTGGTCGTGTGTCTGACTGAGTGAGACGACAAGTTGATGCGACACGGCACTTTCACCCGACGCTCACAATCAGCACAGGGTCTCTATTGAGGGCTCGTCCCAAACCGACCGGGGCGACGCTTCTGGTTCCGTGCCCGCGGCGACCAACAACCGTCGCTGCCCGACCAATGATTGCCACCACGCCCGTCCTTAGCCGACTGAGTGCGACGGAAGCACCAGGGCGCAATTTATTCACTCGCTCGTCACCATATAATACATGGAGAACAGAGGAGGCGGCGCCATCACTACCAAGCACGCAGTGCCGTCCTCCAGACAGAGCCTCTCGGCACAACAAGTGCGTTCAAACAATCAATGGCTCACGAGATTACTCTGCAATTCACACCATAGTATCGCATTATTCTACTACGTTCTTCATCGATGCAAGAGAAGAGATATACCATCTGCAGAGAGTCATATGATTGATTCCGAGGAATATCAGACACATAGGCCTCCCTGCGCACGCCAAGGGGCCCTCTGCCCTCCGAGTCCAATAGTTCCTTAGTTTGTCCATGGTCGCACCGGATACATGGCCAATCTCGCGCCGCCAAGGCATTGGGGTGCGAGGAAAGGAAAACAATCACACCTTCACAAACATCCCATCATTCCATGCTCTGCTCAACAATGAAACTATCCTACGGTCACCTAACGAAACCTTGT
***K. galanga***	785	TGAACTGGGGGGGTGGTCTCGCCTGAGTTGGGGCCACAAGTTGATTGATGGACGTGACGACGTGGCACTTTCGCCCGCCGCCGCAATCAAATCACACAGGGTCTTCTTTCTTCACATGAATTGAGGGCTCACCCCAAAAAGAAGAGGACAATGTTCGGTTCCCGCTCGTGGCGAAACCAACACCCATCGTGCCAGAAGACTTGAGACTACCCGCTCGTCAACGGACTGTGCCCGCCAGAAGGAGGAGGGCACACGGGGCCAATTTACGCGCTCAAGCACCAGCATCGCAGGGAGAAAAGGCGGCAATGCCATGACTAGCAAAGCAGGCGTGCCATCGGCCAAAAGGCCTCGGGCACAACTTGCGATTAAAAGAATCAATGGTTCACGAGATTCTGCAATTCACACAAAGTATCGCATTTCACTACGTTCTTCATAGATGCAAGAGATGCGAGATATCGCATAGCCGAGAGTACATTTGATTCAGAGAAATCTCTGACGCGCTGGGCTCCCCGTGTGCACGTACGCACGGCTGAGGGGCCCTCTGCTTCAGAGTACATTCATTGTTCCTTAGTGCCGATCGCACTGAGCTACGTTGCCATTCCCCCCATCCATCCATCCATCCCACGACAACCATAGTAGAGAGGGGGGGGGGGTGAGATAGAAGCGATGGAGTTTAGGTACGACACAGTCACACAGTGCACAACGGCATCCATCACTTTATTGAATGATCTCGCAAGCAGAGTTCCGCAGCGCGTGCGTACGTAACCAGGGTATGGCCTTTGCGT
***K. elegans***	710	CGACAGAACAATCACACAGGGACTCTTGAGGAATCATCCCGAAACGACGGGGAGCGAATGTGTCTCGTTCCCGACTAACGGTCGACCAACACCCATCGAGCCCGACGAATGCCGATGGCTACACCGCTCTGCGACCGACGGTGCCCGAGGCCAAACGGGGCCAATTTGACGCCGCTATGCACCAAAAGGGAGCAAAGGCCGGAAATGCAAGGACGCCCAAGCAGGCGCTGCCCTCGGCCAGAAGGCATCGGGCCACAACTGGCGTTCAAAGGACTAAAGGGTTCACAAGATTCTGCAATTCACACAAAGTATCGCATTTCACAACGTTCTTAAGCGAGAGCAAGAGCCAAGATAGTCAATTGACTGAGAGTCATTTGATTTGGATGGATGGATTCCGAAGAATCTCTGGCGCATTGGGCTCCCCTTGGGGCACGCACGATGAAGGGGCGGGCCCGCGGCGTCCGAGTTCATTGTTCCTTAGTGCTCATCGCACCGAACTAGCGCTCAATCGAGTCGATCGATAGCCCACCAACACACACGACATGGCATGGCATGGAAGGGATGGAAGTTAGATAGGGATAGGATAGGATAGGAAAGAAAAGAAAGCGACACAATCGTTAACCTCCAATCCATCACTTCAATTATATTCAAGCACGCTCTTTCAACAGACGGTTCCCTACGCAAACCTAGGTAAGACACGTTAACTTTGT
***K. pulchra***	743	GATAGCTTAATTCGGCGGGTGTCTCGCCTGACTGGGGCCACAAGTTGATGGGCGCGACGGCACTCAACGTGCCCGACGCCGCAATCACACAGGGTCTCTTGAGGACTCATCCCGAAACGACGGGGACGATGTTCTGTTCCCGCTCATGGCGACCAACACCCATCGTGCCCGACGAATGCCGATGGCTACCCGCTCTTCGACCGACTGTGCCCGAGGGCACACGGGGCCAATTTACGCGCTCTGCACCAGCATGGAGCAAAGGCGGCAATGCCATGACTCCCAAGCAGGCGTGCCCTCGGCCAGAAGGCCTCGGGCACAACTTGCGTTCAAAGACTCAATGGTTCACGAGATTCTGCAATTCACACCAAGTATCGCATTTCACTACGTTCTTCATCGATGCAAGAGCCGAGATATCCATTGCCGAGAGTCATTTGATTGATTGATTGATTGATTCCAAACAATCTCTGGCGCATTGCGCTCCCCTTGTGCACGCACGATAAGGGGCCGACCCTCCGCTTCAGAGTTCATTGTTCCTTAGTACTGATCGCACCGAGCTACGGTCAATCGATCGATCGATCGATCGCCCACCAACATACATGGCATGGCATGGATGGATGGATGGGAGATAGATAGATAGATAGATAGATAAGAAAGGGACACATTCACACGTTCACAACCATCCATCATTCTATTATGCTCTCTCAACAATGATCCTTCCGCAGGTTCACCTATAGAAACCTTGT
***K. parviflora*****(M)**	676	TTGTTGAGAGAGCACATAACAGAAGGATGGATGGTGGCGAACGAGTGAATGTGTCCCTTTCCTCCGCCCCTCGACAAGCAAGCATGCATGCATGCAGGTCGGTCGGTGGACGATTCGACCGTTCGTTGCTACCGATCAGCAAGAAGGAACAAACAACTAAGAAGCAGAGGGCTCGTCGGGGGGAGCAAGCAGATCACCGATGCATAGAATGATTCCTAATCAAACAATCAAAAAGAATCTCGGCAACAAACAATTCTGTCTCTTGCTTCATTGATGAATGAAGTAAAAAACTAGATTTGATGTGTGTTATAGAATAGAACATTGTATTGCATCAATTATTTAATCTGTCAGCAAGTAGGGCCCATGCCGTCCTGCCGGACGGCAGCCCGGCCTGCCTAGTCATGACTTTTATGCACTCCCCTCCACCCCACCCTGCGATGGTGCTGAGGGATGAGATTACGTTGACCCCGTCCCGTCCTGCCCTCAGCCGACAGACGAACGACCAGTCGCTCAGTCGTCGGTCGGCAGGAGTGATTGGTGGTTGATCGGATACAGAACAGCAAATCGTCCATCTCCTCTCGTCAGACGACCAACCAACCAACGACCATTTTACCGTCATGGCGGCGTCGGGCGAAAGTGCCGTAGGCACCTCTCATCGATCAACTATCGTGCCCCA
***K. parviflora*****(T)**	651	TTGCGAACGTGTGAATGTGTCCCTTTCCTTTGCCCCTCGACGTGCAAGCATGCATGCATGCATGTCGGCGGGGGATTAATCACCGTTCGTTGCTATCATCAGTACTAAAGAATGATGACCTAAGAAACGGACGCCTCGTCGTGGGGCGCGAGCCCACCGCTGCGTAGATTATTCCTAATAAATCAACAATAATCATGGCAATTGTTGTTTCTTCTCTCGCTTCAATCATTAATGAAGTGAATTACTAGATTTGATGTGTGTTATTTAATGAACTATTGCATCAATTATTTCATCTAAGGTCGTAGGCCATGCCTTCTGCCTTACGGCAGACCGGCTTCCTAGTCATTACTTTGTCGCCTCCGCTCTACGCTATGCTTGTTCTTAGGGCACAAGTTAGATCGATGGACCCCTCCCGTCAGGCACTAGTACGCCCGACGACCGACCAGTCACCAGGCGTCGGTCGGCATCGAAGGATGGTCGGGAGATCTGAAAGAGAACAGCAAATTCTGTCCATCTCCTCATCTCAGACGAACGATCACCCGACAAAATCGTGATCGACCGATTCCAGCGACCGTACCTCCTCCCTTCGGCTCGATCAGTGGCAGAGGTGCCGCCACGCGTCAGACAATCTACCCGCCCGACCACTTCACC
***K. marginata***	714	GATATGCTTAAACTCGGCGGGTGGTCTCGCCTGACTTGGGGCCACAAGTTGATGGGCGCGACGGCACTCAACGTGCCCGACGCCGCAATCACACAGGGTCTCTTGAGGACTCATCCCGAAACGACGGGGACGATGTTCTGTTCCCGCTCATGGCGACCAACACCCATCGTGCCCGACGAATGCCGATGGCTACCCGCTCTTCGACCGACTGTGCCCGAGGGCACACGGGGCCAATTTACGCGCTCTGCACCAGCATGGAGCAAAGGCGGCAATGCCATGACTCCCAAGCAGGCGTGCCCTCGGCCAGAAGGCCTCGGGCACAACTTGCGTTCAAAGACTCAATGGTTCACGAGATTCTGCAATTCACACCAAGTATCGCATTTCACTACGTTCTTCATCGATGCAAGAGCCGAGATATCCATTGCCGAGAGTCATTTGATTGATTGATTGATTCCGAAGAATCTCTGGCGCATTGGGCTCCCCTTGTGCACGCACGATGAGGGGCGGACCCTCTGCTTCCGAGTTCATTGTTCCTTAGTGCTGATCGCACCGAGCTACGGTCAATCGATCGATCGATCGCCCACCAACATACATGGCATGGCATGGATGGATGGGAGATAGATAGATAGATAGATTAAGAAAGGGACACATTCACACGTTCACAACCATCCATCACTCTATTATGCTCTCTCAAGAGAGATCCTTCCGGGATGT

**Table C1:** Qualitative and qualitative scoring of morphological characters for *Kaempferia* species.

Morphological traits (vegetative)	Measurement (Scoring)
Plant height	Tall >21 cm (1), Short <21 cm (0)
Number of leaves	Many >5 (1), Few <5 (0)
Leaf length	Long >21 cm (1), Short <21 cm (0)
Leaf width	Wide >9 cm (1), Narrow <9 cm (0)
Leaf petiole length	Long >9 cm (1), Short <9 cm (0)
Leaf petiole width	Wide >1 cm (1), Narrow <1 cm (0)
Number of leaf scale	Many >1.7 (1), Few <1.7 (0)
Leaf scale length	Long >7 cm (1), Short <7 cm (0)
Leaf scale width	Wide >1.7 cm (1), Narrow <1.7 cm (0)
Rhizome diameter	Wide >1.18 cm (1), Narrow 1.18 cm (0)
Plant habit	Erect (2), Semi adpressed (1), Adpressed (0)
Leaf shape	Elliptic (2), Lanceolate (1), Ovate (0)
Leaf apex	Acute (1), Non- acute (0)
Leaf base	Auricultae (4), Cuneate (3), Cordate (2), Rounded (1), Unequal (0)
Leaf margin	Undulated (1), Non-undulated (0)
Lear variegation	Present (1), Absent (0)
Leaf adaxial colour	Green (2), Yellow green (1), Grey green (0)
Leaf abaxial colour	Green (2), Yellow green (1), Grey green (0)
Leaf Petiole colour	Green white (3), Green (2), Yellow green (1), Grey green (0)
Leaf Scale colour	Red (1), Green (0)
Leaf margin colour	red margin (1), non-red margin (0)
Rhizome shape	Globose (1), non-globose (0)
Rhizome external colour	Yellow (4), Brown (3), Green yellow (2), Grey orange (1), Grey Brown (0)
Rhizome internal colour	Grey Yellow (4), Yellow (3), Yellow orange (2), Dark Purple (1), Light purple (0)

**Fig. D1:**
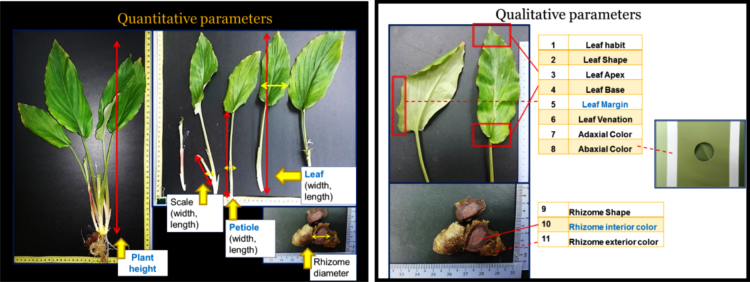
Qualitative and quantitative measurement technique of *Kaempferia* species.

**Table E1:** Molecular markers used in phylogenetic studies of Zingiberaceae.

Num	Species, genus or family	Molecular markers used	Reference
1	*Boesnbergia*	*petA-psbJ* (chloroplast DNA),	[12]
2	*Hedychium*	ITS (nrDNA)	[13]
3	*Alpinia*	*matK* (chloroplast) & ITS (nrDNA)	[8]
4	*Globba*	*trnK*-*matK* (chloroplast) & ITS (nrDNA)	[14]
5	*Amomum*	*matK* (chloroplast) & ITS (nrDNA)	[15]
6	*Curcuma*	*trnL-trnF, psbA-trnH, matK* & ITS (nrDNA)	[16]
7	Zingiberaceae	*trnL-F* & ITS (nrDNA)	[17]
8	Zingiberaceae	*matK* (chloroplast)	[18]

## Appendix A

See Fig. A1

## Appendix B

See Table B1

## Appendix C

See Table C1

## Appendix D

See Fig. D1

## Appendix E

See Table E1
