# Supplementary material for: Morphological and molecular datasets for Kaempferia species
Source: Data Brief. 2018 Oct 27;21:1678–85. doi: 10.1016/j.dib.2018.10.097 (PMC6247442; doi:10.1016/j.dib.2018.10.097)
Supplement: Supplementary file 1 — Supplementary material [file mmc1.docx]

Conflict of interest statement

Article title: Identification of ethnomedicinally important *Kaempferia* L. (Zingiberaceae) species based on suitable DNA region and morphological traits

The authors whose names are listed immediately below certify they have NO affiliations with or involvement in any organization or entity with any financial interest (such as honoraria; educational grants; participation in speakers’ bureaus; membership, employment, consultancies, stock ownership, or other equity interest; and expert testimony or patent-licensing arrangements), or non-financial interest (such as personal or professional relationships, affiliations, knowledge or beliefs) in the subject matter or materials discussed in this manuscript.

Author(s) names Signature


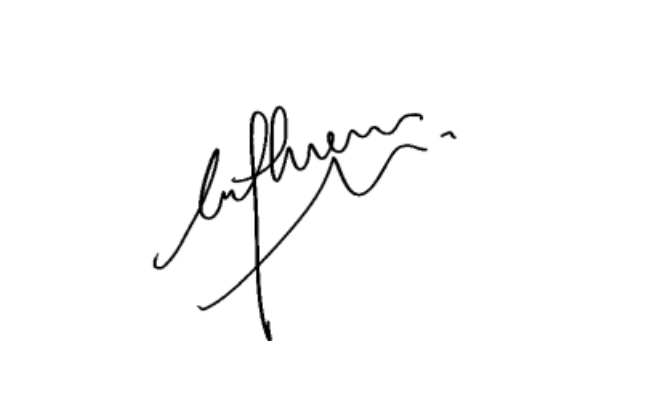


1. Catherine Labrooy

PhD candidate

Department of Crop Science, Faculty of Agriculture,

University Putra Malaysia, 43400 Serdang,

Selangor Darul Ehsan Malaysia

Email: [catherinelabrooy@gmail.com](mailto:catherinelabrooy@gmail.com)

1. Thohirah Lee Abdullah


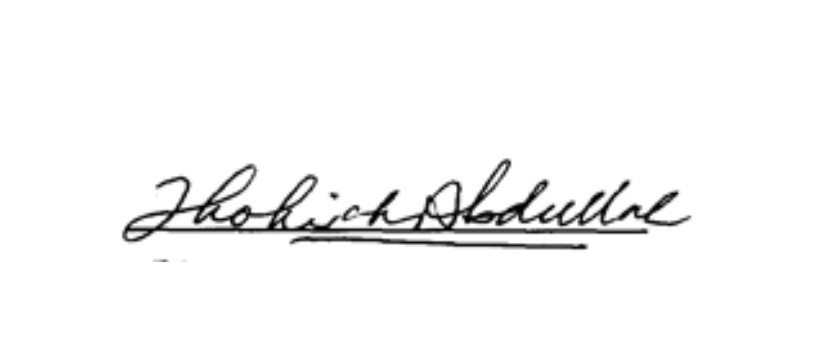


Associate Professor and lecturer

Department of Crop Science, Faculty of Agriculture,

University Putra Malaysia, 43400 Serdang,

Selangor Darul Ehsan Malaysia

Email: [thohirah@upm.edu.my](mailto:thohirah@upm.edu.my)

1. Johnson Stanslas


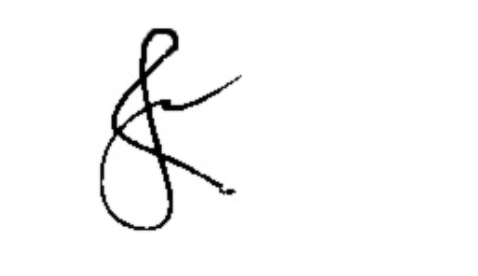


Professor and lecturer

Department of Medicine,

Faculty of Medicine and Health Sciences,

University Putra Malaysia,

43400 Serdang, Selangor Darul Ehsan Malaysia

Email: rcxjs@upm.edu.my
